# Novel *Anaplasma* Variants in Small Ruminants From Central China

**DOI:** 10.3389/fvets.2020.580007

**Published:** 2020-11-26

**Authors:** Yan Zhang, Yanyan Cui, Yanting Sun, Huiyuan Jing, Changshen Ning

**Affiliations:** ^1^College of Animal Medical Science, Henan University of Animal Husbandry and Economy, Zhengzhou, China; ^2^Zhengzhou Key Laboratory of Veterinary Biological Products Technology, Zhengzhou, China; ^3^College of Biology and Food, Shangqiu Normal University, Shangqiu, China; ^4^College of Animal Science and Veterinary Medicine, Henan Agricultural University, Zhengzhou, China

**Keywords:** phylogenetic analysis, 16S rRNA, variants, *Anaplasma capra*, *gltA*

## Abstract

*Anaplasma capra* is an emerging zoonotic pathogen that pose a risk to the health of human and veterinary animal. Numerous variants in a variety of domestic and wild animals had been reported since its discovery and confirmation in humans in 2015 and its first detection from goat blood during 2012–2013. In order to find out more *A. capra* variants data of *A. capra* in central China, 16S rRNA, *gltA, groEL*, and *msp4* genes of this pathogen were amplified from sheep and goat samples collected during 2011–2015 and phylogenetic analysis of these sequences were conducted. The results of 16S rRNA and *gltA* manifested that partial sequences obtained in this study were 100% identical with *A. capra* isolates, while phylogenetic analysis results of *groEL* and *msp4* showed that the obtained sequences were independent with all other *Anaplasmas*, formed separate branches on the evolutionary trees. What needed to be emphasized was that the 16S rRNA and *gltA* gene sequences of X51 (KX505302 and KX450269), a sample from Shandong in 2011, were found to be 100% identical with *A. capra*. Therefore, we could speculate that the occurrence of *A. capra* may be earlier than its first discovery and report. And the *A. capra* isolates in central China were novel variants which were different from known genotypes.

## Introduction

*Anaplasma* genus members are zoonotic pathogens with remarkable importance in both human and veterinary health. Three *Anaplasma* species have so far been identified that infect human beings. The first discovered and the most widespread is *Anaplasma phagocytophilum*, which was first reported in 1994 in Wisconsin, USA, and caused a febrile illness (1). Subsequently, the disease was named human granulocytic anaplasmosis based on its main symptoms. In China, since the first HGA case was reported in Anhui province in 2006 (2), more than 90 HGA cases have been reported in Beijing, Tianjin, Shandong, Hubei, Henan, and the Inner Mongolia Autonomous Region (3). In 2007, a patient from Cyprus was diagnosed as infected with *A. ovis* (4); but no other human infection with *A. ovis* have been reported. *Anaplasma capra*, a novel *Anaplasma* member, was first identified in 28 patients from Heilongjiang province in 2015. These patients had symptoms of high fever, headache, malaise, dizziness, and muscle pain (5). Since then, numerous reports on *A. capra* infections in various ruminants and wild animals have been published (6–9). The widespread distribution and genetic diversity of the novel identified *Anaplasma* species mentioned above highlight the dangers of tick-borne disease caused by the *Anaplasma* genus.

In previous studies, *A. capra* DNA fragments were reported to be detected in domestic animals and some species of wild animals and ticks (6–10). More importantly, various genotypes of this pathogen and *A. capra*-like bacteria were documented, a finding similar to the case of *A. phagocytophilum* where its many genetic variants result in poor immunity from the vaccine based on it. In the present study, we originally planned to amplify the almost full length of *Anaplasma* 16S rRNA, *gltA, groEL*, and *msp4* genes from *Anaplasma*-positive sheep and goats DNA samples, which was only confirmed to be positive for *Anaplasma* based on the short fragment of 16S rRNA gene (11) and to analyze the genetic difference of *Anaplasma*, especially that of *A. phagocytophilum*, but the results were unexpected. The sequence alignment results showed that some of the sequences we obtained had higher homology with *A. capra*, which may be new *A. capra* isolates or genotypes.

## Methods

### Ethics Statement

This study was conducted in accordance with the Chinese Laboratory Animal Administration Act of 1988. The research protocol was reviewed and approved by the Research Ethics Committee of Henan University of Animal Husbandry and Economy. The field studies did not involve endangered or protected species.

### Specimens and DNA Samples

Altogether, 212 DNA samples obtained from sheep and goats blood and was PCR-screened to be positive for *Anaplasma* using primer pair EE1 and EE2 (12) was used in the present study. All DNA samples were stored at −20°C until use.

### PCR Amplification

Conventional PCR was used to amplify a 1,133-bp fragment of the 16S rRNA gene from *Anaplasma*. The primer pair was from two previous studies (13, 14). Nested PCRs specific for three protein coding genes, citrate synthase (*gltA*), heat shock protein (*groEL*), and major surface protein 4 (*msp4*), were conducted using previously described primers and PCR conditions (Supplementary Table 1). PCR amplification was performed as follows: 1.0 μL of extracted DNA was added to a 25 μL PCR mixture containing 2.5 μL of 10× PCR buffer (Mg^2+^Plus), 2.0 μL of dNTPs, 1.0 U of Taq DNA polymerase (LA Taq for the first round, rTaq for the second round) (Takara, Dalian, China), 1.0 μL of each primer (10 pmol), and 16.5 μL of distilled water. Reactions were conducted in an automated DNA C1000 thermal cycler (Bio-Rad, Beijing, China). Each PCR reaction was conducted at least twice using nuclease-free water as the negative control in each reaction, whereas a sheep DNA sample that was preserved in the parasitology laboratory of Henan Agricultural University and previously verified as *Anaplasma*-positive was used as the positive control. PCR products were visualized by UV transillumination on a 1.0% agarose gel followed by electrophoresis and staining with GelRed™ (Biotium Inc., Hayward, CA, USA).

### DNA Sequencing and Phylogenetic Analysis

Positive PCR products were purified using Montage PCR filters (Millipore, Bedford, MA). The products were sequenced using the BigDye Terminator v 3.1 cycle sequencing kit (Applied Biosystems, Foster City, CA) on the ABI 3730 DNA analyzer (Applied Biosystems). The sequence accuracy was confirmed by two-directional sequencing and by sequencing a new PCR product when necessary. The obtained sequences were analyzed by a BLAST search of the GenBank database (http://blast.ncbi.nlm.nih.gov/Blast.cgi). Sequence assembly, phylogenetic analyses and evolutionary analyses were performed using Mega X software (http://www.megasoftware.net/). The number of base differences per site from averaging over all sequence pairs between groups were shown as Supplementary Tables 5–8 for each gene. These analyses involved 25 nucleotide sequences for 16S rRNA gene, 23 for *gltA* gene, 21 for *groEL* gene and 23 for *msp4* gene, respectively. All positions containing gaps and missing data were eliminated (complete deletion option). There were a total of 1,314, 487, 328, and 366 positions for each gene in the final dataset. Phylogenetic trees were constructed using the neighbor-joining algorithm method (15) with the Kimura two-parameter model for nucleotide sequence analysis. To examine the effect of the method of analysis on the resulting phylogeny, maximum parsimony analyses were also conducted. The stability of the trees obtained was estimated by bootstrap analysis with 1,000 replicates.

### Nucleotide Sequence Accession Numbers

The representative sequences obtained in this study were deposited in the GenBank database under the following accession numbers: 16S rRNA (KX505293–KX505303), *gltA* (KX450266–KX450272), *groEL* (KX388350–KX388358), and *msp4* (KX528101–KX528113).

## Results

### Phylogenetic Analysis of 16S rRNA and *gltA* Genes

**Figure 1:**
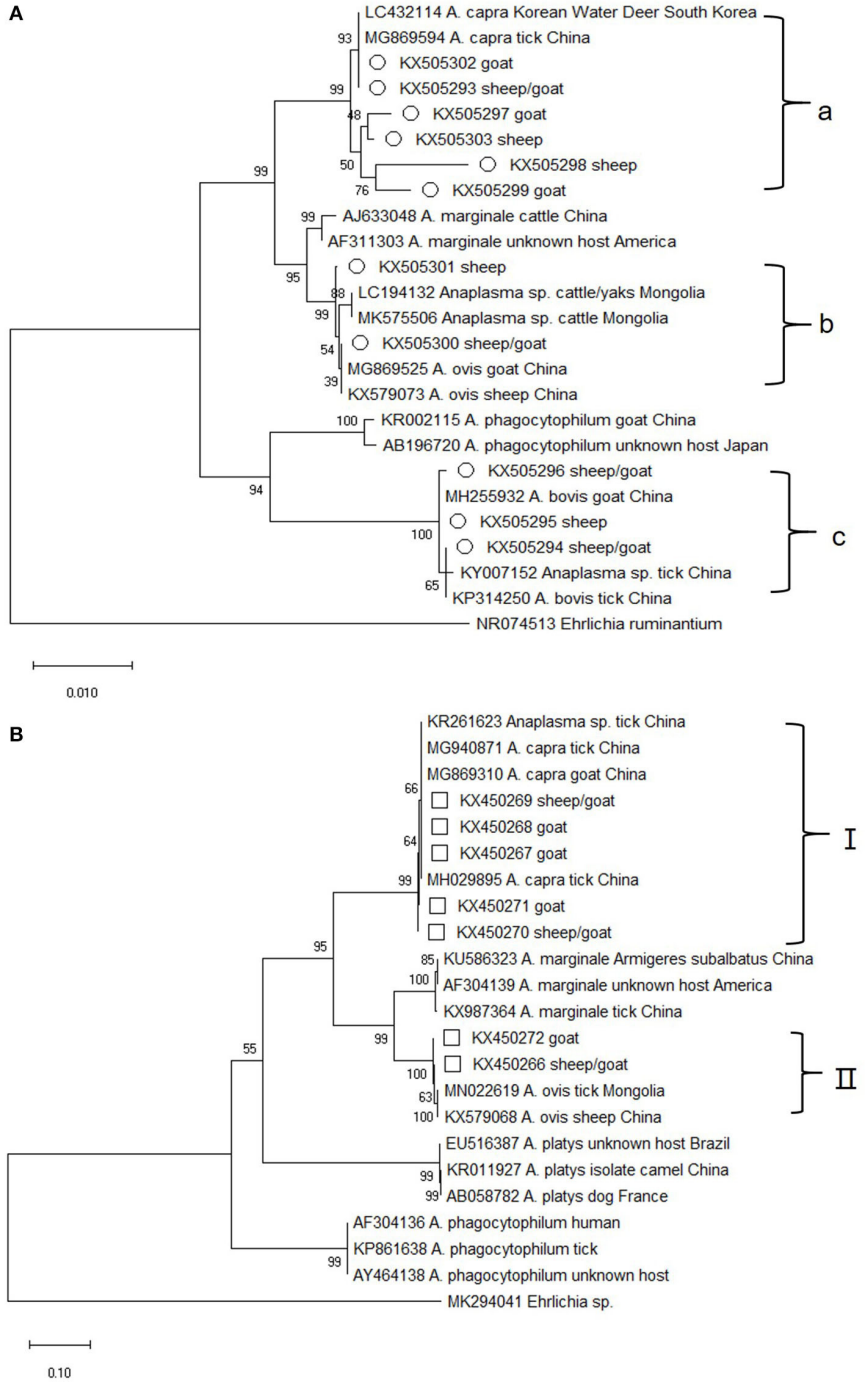
Phylogenetic tree of *Anaplasma* based on 16S rRNA and *gltA* gene. An alignment of 1,133 bp 16S rRNA gene sequences **(A)** and 759 bp *gltA* gene sequences **(B)** was used to construct the neighbor-joining phylogenetic tree using Kimura two-parameter model in Mega X. *Ehrlichia ruminantium* or *Ehrlichia* sp. was used as the outgroup. The sequences marked with hollow circle and hollow box were obtained in the present study.

Of the 212 *Anaplasma*–positive specimens, the 16S rRNA (~1,133 bp) gene was identified in 30. Sequence analysis showed that 11 16S rRNA sequence types, which grouped into three groups on the evolutionary tree, were obtained (Figure 1A). The sequences in group a shared 98.7–100% similarity with *A. capra* isolates from China (MG869594) and Korea (LC432114), but were distinct from other *Anaplasma* species. They represented 70% (21/30) of all the 16S rRNA genes amplified from the sheep and goat blood samples collected from several Chinses provinces between 2011 and 2015 (Supplementary Table 2). KX505300 and KX505301 were closely related to *A. ovis* isolates (MG869525 and KX579073, with 99.8–100% similarity) and formed a clade (group b). The sequences in group c shared 99.0% identity with the sheep and goat isolates of *A. bovis* from China.

Altogether, 118 of the 212 blood samples from sheep and goats were positive for the *gltA* gene. Sequence analysis showed that seven sequence types, which fell into two distinct groups, were obtained. The group I sequences, which represented nearly all of the *gltA* sequences (96%, 113/118) in this study, were highly homologous (99.6–100%) to *A. capra* isolates from China (MG940871, MG869310, and MH029895) and appeared on a separate evolutionary branch with them (Figure 1B). It is worth mentioning that the sequences from this group corresponded to those in group a of 16S rRNA gene (the two gene sequences could be amplified at the same time in 9 blood samples) (Supplementary Table 3). The group I specimens were collected in 2011–2015 (Supplementary Table 4), which is same as that for 16S rRNA. The remaining *gltA* sequences along with two *A. ovis* sequences from China formed group II, which was similar with the 16S rRNA gene sequences in group b. The sequences in group II shared a similarity of 99.3–99.9%.

### Phylogenetic Analysis of the *groEL* Gene

**Figure 2:**
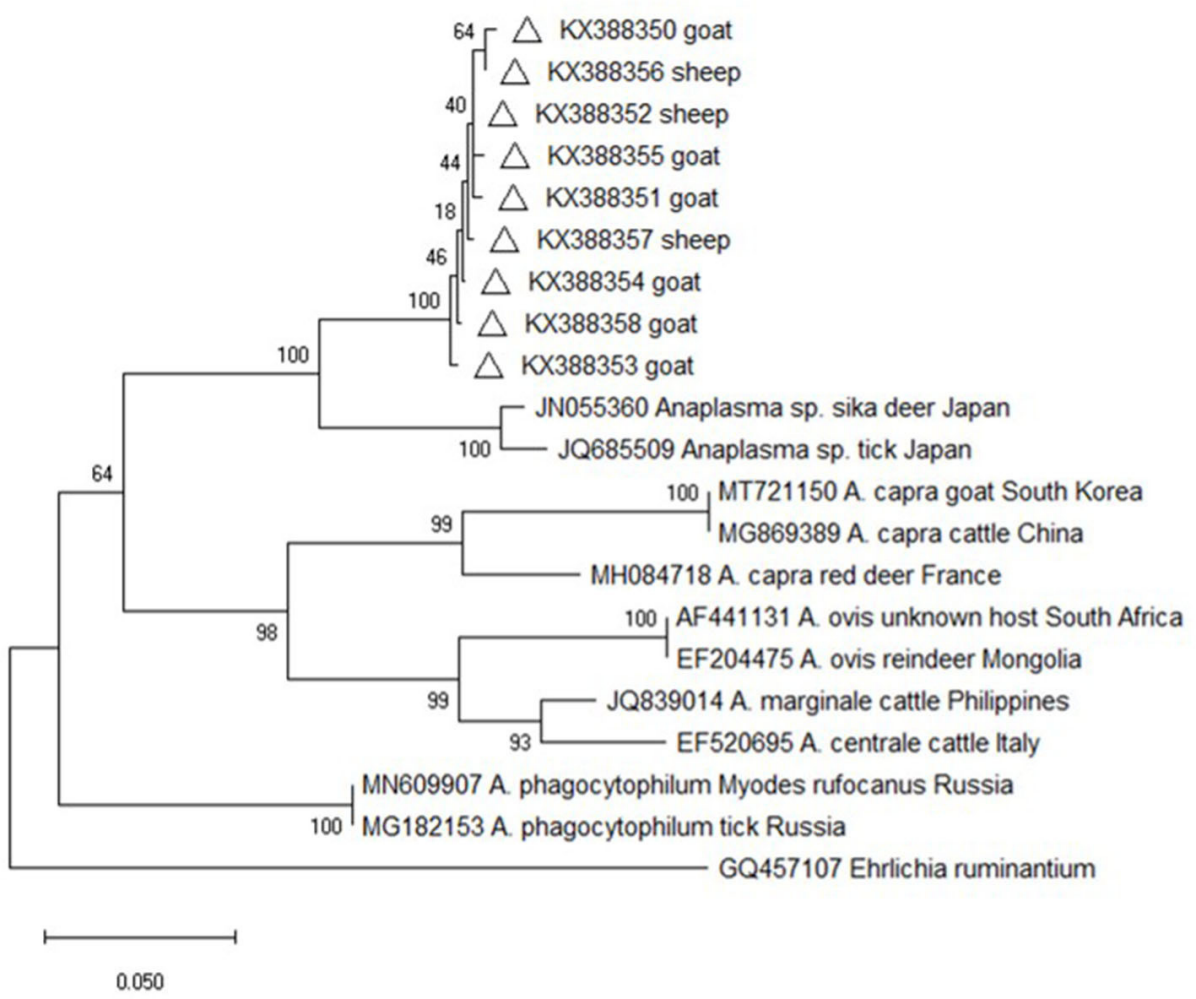
Phylogenetic tree of *Anaplasma* based on *groEL* gene. An alignment of 341 bp *groEL* gene sequences was used to construct the neighbor-joining phylogenetic tree using Kimura two-parameter model in Mega X. *Ehrlichia ruminantium* was used as the outgroup. The sequences marked with hollow triangle were obtained in the present study.

Altogether, 162 *groEL* gene sequences were amplified from the *Anaplasma*–positive DNA samples. Although the sequence alignment analysis showed that nine different sequence types contained 1–7 nucleotides difference, the sequences were almost identical at amino acid level (data not shown). Furthermore, the online sequence alignment showed that the obtained sequences were distinct with those of other known *Anaplasma* species and strains, with the highest similarity (90.0%) with an *Anaplasma* sp. isolate from deer in Japan (JN055360). Phylogenetic analysis based on the *groEL* gene sequences also revealed that the isolates from this study were evolutionarily distant from other *Anaplasma* species and strains and formed an independent branch on the evolutionary tree, which was supported by high bootstrap values (100%) (Figure 2).

### Phylogenetic Analysis of the *msp4* Gene

**Figure 3:**
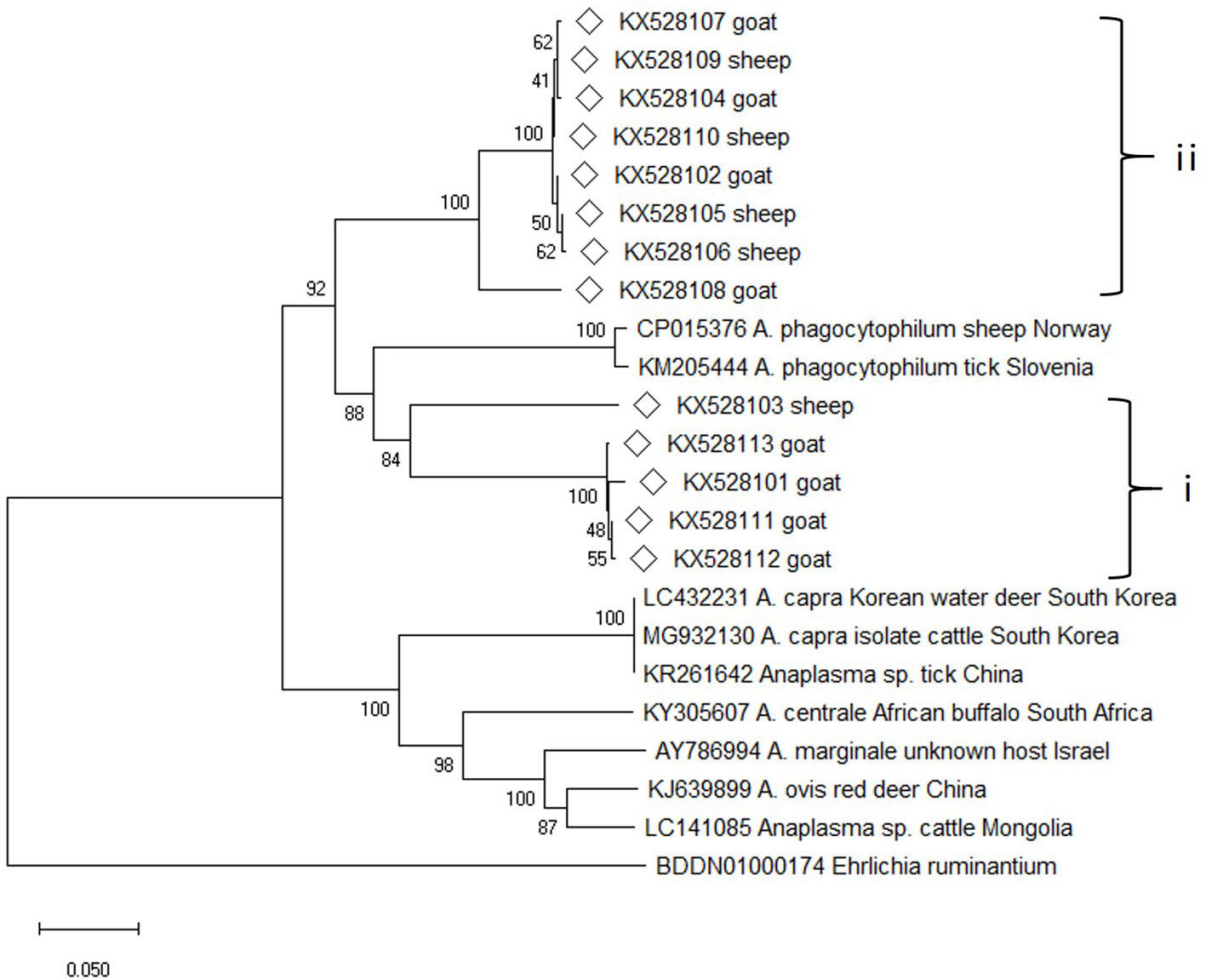
Phylogenetic tree of *Anaplasma* based on *msp4* gene. An alignment of 350 bp *msp4* gene sequences was used to construct the neighbor-joining phylogenetic tree using Kimura two-parameter model in Mega X. *Ehrlichia ruminantium* was used as the outgroup. The sequences marked with hollow diamond were obtained in the present study.

Altogether, 54 *msp4* gene sequences were amplified from the DNA samples, and the sequence alignment analysis conducted on them showed that 13 sequence types were present. The pairwise sequence alignments revealed that the sequences fell into two groups (group i and group ii) with a similarity score of 98.4–99.7% within each group, and a score of 71.8–76.0% between the groups. The online sequence alignment results showed that the sequences obtained in this study differed greatly from the known sequences, and the highest similarity score (75.7% for group i and 75.0% for group ii) was seen with two *A. phagocytopuilum* isolates (CP015376 and KM205444). Phylogenetic analysis based on the *msp4* gene sequences also revealed that the isolates from the present study were distinct from other well-defined *Anaplasma* species and fell into two separate clades on the evolutionary tree (Figure 3).

## Discussion

In 2015, Li et al. identified a novel tick-borne *Anaplasma* species they called “*A. capra*” (5). Furthermore, the unknown bacterium was able to be propagated in HL-60 cells, which mainly allow for the growth of human isolates. These results verified their former findings that the agent was a new species of *Anaplasma* genus with zoonotic potential, just like *A. phagocytophilum*. They also mentioned that 37 blood samples from asymptomatic goats were positive for an unknown *Anapalsma* species during 2012–2013. Following its first report as a human pathogen in 2015, *A. capra* has been detected in several tick species, including *Ixodes persulcatus, Haemaphysalis longicornis, H. qinghaiensis*, and *Rhipicephalus microplus* (5, 10, 16, 17), in sheep and goats (9), in cattle (18), in dogs (19), in Korean water deer (20). These findings together with the results obtained herein suggest that additional domestic and wild animals and/or tick species might be the hosts and tick vectors of *A. capra* and are involved in its transmission and maintenance, a possibility the needs further investigation.

In the present study, 16S rRNA and *gltA* gene fragments were also PCR-amplified from the samples from sheep and goats collected in 2012–2015, and even from the blood samples collected in 2011, which is earlier than the first literature to report the origin of this bacterium. Therefore, we can speculate that the novel species may have existed in animals/ticks for a long time and had either not been detected or was mistaken for other related species. Our results showed that the *gltA* sequences in group I were 100% identical to another *Anaplasma* sp. isolate (KJ700628) and share 99% sequence identity with an *A. centrale* isolate (AF304141). In 2018, Khumalo et al. reported a similar finding and confirmed that the *A. centrale* 16S rRNA gene sequence (Aomori strain, AF283007) from cattle in Japan that was acquired in 2001 was actually *A. capra* (21).

As shown in Supplementary Table 3, the 16S rRNA sequences in group a and the *gltA* sequences in group I could correspond to each other. The two genes from nine of the samples were both closely related to the human *A. capra* pathogen and group in a separate clade with other *A. capra* isolates, but were distinct from other *Anaplasma* species, indicating the novelty of this pathogen (Figure 1). In addition, sequences in 16S rRNA group b and *gltA* group II were both closely related to *A. ovis*, although these two sets of sequences could not correspond to a same sample. This result along with the fact that KX505294–KX505296 obtained in this study were grouped with *A. bovis* isolates from China suggested that there may be new *A. bovis* and *A. ovis* isolates in the samples collected.

Nevertheless, the *groEL* and *msp4* gene sequences from this study were distinct from all other known *Anaplasma* species and strains with each having low sequence similarity values (90% for *groEL* and 74.6% for *msp4*), indicating the possibility of novel *Anaplasma* genotypes or species in sheep and goats in China and suggesting a high degree of genetic diversity and host tropisms in *Anapalsma*, as has already been documented for *A. phagocytophilum* (22). Because it's unclear whether these new genotypes or species vary in their pathogenicity profiles toward non-human and human animals, these profiles warrant further investigation.

In summary, novel *Anaplasma* genotypes or species closely related to human *A. capra* pathogen were identified with divergent *groEL* and *msp4* genes in sheep and goats in central China. Further studies should be conducted to fully elucidate the host range, vector ticks, pathogenicity characteristics and geographic distribution of this bacterium.

## Data Availability Statement

The datasets presented in this study can be found in online repositories. The names of the repository/repositories and accession number(s) can be found in the article/Supplementary Material.

## Ethics Statement

The animal study was reviewed and approved by Research Ethics Committee of Henan University of Animal Husbandry and Economy. Written informed consent was obtained from the owners for the participation of their animals in this study.

## Author Contributions

CN conceived the study. CN and YZ designed the experiments. YZ and YC performed the experiments. YZ, YS, and HJ performed data analysis. HJ and CN wrote the manuscript. All authors approved the final version of the manuscript.

## Conflict of Interest

The authors declare that the research was conducted in the absence of any commercial or financial relationships that could be construed as a potential conflict of interest.
