# Supplementary material for: Novel Anaplasma Variants in Small Ruminants From Central China
Source: Front Vet Sci. 2020 Nov 26;7:580007. doi: 10.3389/fvets.2020.580007 (PMC7732622; doi:10.3389/fvets.2020.580007)
Supplement: Supplementary file 1 [file Data_Sheet_1.docx]

Table S1 The primers used to amplified 16S rRNA*、msp4*、*gltA* and *groEL* genes

| Target gene | Primer name | Nucleotide sequence 5’-3’ | Product size | Annealing temperature | References |
| --- | --- | --- | --- | --- | --- |
| 16S rRNA | 16S-f  16S-r | GCGATTTTAGAGTGYGGAGATTG  TACAATACCGGAGTAAAAGTCAA | 1133bp | 56℃ | (Wen et al., 2002; Li et al., 2012) |
| *msp4* | Ap-msp4-1F  Ap-msp4-1R | TCCCTTTTAATTAGTGGATCCTCAT  CATACTGTTCATCGAAAATTCCGTG | 849bp | 52℃ | (de la Fuente et al., 2005) |
|  | msp4f | CTATTGGYGGNGCYAGAGT | 350bp | 54℃ | (Bown et al., 2007) |
|  | msp4r | GTTCATCGAAAATTCCGTGGTA |  |  |  |
| *gltA* | Outer-f  Outer-r | GCGATTTTAGAGTGYGGAGATTG  TACAATACCGGAGTAAAAGTCAA | 1078bp | 55℃ | (Li et al., 2015) |
|  | Inner-f  Inner-r | GGGTTCMTGTCYACTGCTGCGTG  TTGGATCGTARTTCTTGTAGACC | 794bp | 55℃ |  |
| *groEL* | GroESL1F  GroESL1R | TATAGCTAGCATAATTACCCAGAGC  GGTTAGTTCTGCTTTCGATGC | 842bp | 53℃ | (Zhang, 2015) |
|  | GroESL2F  GroESL2R | TTATGTCTATGCGCCGTG  CGGACCTTGCCACATTTT | 339bp | 51℃ |  |

Table S2 Sampling time and sites of the obtained 30 16S rRNA gene sequences

| Accession No. | Sequences | Sampling time (year) | Sampling sites |
| --- | --- | --- | --- |
| KX505293 | MP11 | 2015 | Guizhou |
|  | YY1, YY3, YY4, YY9, YY11, YY12, YY15, YY18, YY19, YY20, YY26, LY8 | 2014 | Henan and Shaanxi |
|  | X56, X61, X63 | 2012 | Henan and Shanxi |
| KX505297 | YY13 | 2014 | Henan |
| KX505298 | YY14 | 2014 | Henan |
| KX505299 | YY35 | 2014 | Henan |
| KX505302 | X51 | 2011 | Shandong |
| KX505303 | yang3 | 2014 | Henan |
| KX505300 | MP4，MP40 | 2015 | Guizhou |
|  | X64 | 2012 | Shanxi |
| KX505301 | X59 | 2012 | Shanxi |
| KX505294 | YY5, YY31 | 2014 | Henan |
| KX505295 | YY16 | 2014 | Henan |
| KX505296 | YY29, YY30 | 2014 | Henan |

Table S3 Samples which had 16S rRNA sequences from cluster a and the *gltA* sequences in cluster Ⅰ at the same time

| Sample name | *16S rRNA* gene accession No. | *gltA* gene accession No. |
| --- | --- | --- |
| YY1 | KX505293 | KX450269 |
| YY3 | KX505293 | KX450270 |
| YY9 | KX505293 | KX450269 |
| X61 | KX505293 | KX450270 |
| X63 | KX505293 | KX450270 |
| YY14 | KX505298 | KX450270 |
| YY35 | KX505299 | KX450269 |
| X51 | KX505302 | KX450269 |
| yang3 | KX505303 | KX450270 |

Table S4 Sampling time and sites of the obtained 118 *gltA* gene sequences

| Accession No. | Sequences | Sampling time (year) | Sampling sites |
| --- | --- | --- | --- |
| KX450267 | Y16 | 2015 | Henan |
| KX450268 | G27 | 2015 | Guizhou |
| KX450269 | Y4, Y6, Y9-Y12, Y18, Y20, G22, G23-G26, G28-G31, G34, G37-G39 | 2015 | Henan and Guizhou |
|  | yang1, yang2, YY1, YY9-YY13, YY21-YY24, YY32, YY34- YY39，LY1, LY2, LY6, LY7, LY9, LY11, LY13-LY15, LY17- LY19, LY21-LY25, LY27-LY47 | 2014 | Henan and Shaanxi |
|  | X12 | 2013 | Henan |
|  | X51 | 2011 | Shandong |
| KX450270 | Y2, Y3, Y13, Y14 | 2015 | Henan |
|  | LY5, yang3, YY2, YY3, YY5-YY8, YY14, YY15, YY17-YY20, YY25, YY26, YY29, YY31 | 2014 | Henan and Shaanxi |
|  | X11, X18, X55, X61, X63 | 2012 | Henan and Shanxi |
| KX450271 | G36 | 2015 | Guizhou |
| KX450266 | Y21, G32 | 2015 | Henan and Shaanxi |
|  | LY20 | 2014 | Shaanxi |
| KX450272 | Y17, Y19 | 2015 | Henan |

Table S5 Estimates of evolutionary divergence over sequence pairs between groups of 16S rRNA gene

| Group | P distance |  |  |  |  |  |  |  |  |
| --- | --- | --- | --- | --- | --- | --- | --- | --- | --- |
| Group a |  |  |  |  |  |  |  |  |  |
| Group c | 0.042491 |  |  |  |  |  |  |  |  |
| Group b | 0.018645 | 0.036657 |  |  |  |  |  |  |  |
| *A. bovis* ^*^ | 0.042364 | 0.000634 | 0.036530 |  |  |  |  |  |  |
| *Anaplasma* sp. | 0.027693 | 0.025199 | 0.013825 | 0.024987 |  |  |  |  |  |
| *A. capra* | 0.004693 | 0.042364 | 0.014840 | 0.042237 | 0.025114 |  |  |  |  |
| *A. ovis* | 0.019026 | 0.036276 | 0.000381 | 0.036149 | 0.013445 | 0.015221 |  |  |  |
| *A. phagocytophilum* | 0.034754 | 0.027524 | 0.031963 | 0.027397 | 0.030568 | 0.034627 | 0.031583 |  |  |
| *A. marginale* | 0.016743 | 0.037037 | 0.005708 | 0.036910 | 0.016743 | 0.012938 | 0.006088 | 0.032344 |  |
| Outgroup ^#^ | 0.078640 | 0.083714 | 0.074201 | 0.083333 | 0.078133 | 0.076865 | 0.074581 | 0.077626 | 0.073059 |

^*^ All *A. bovis* gene sequences in the phylogenetic tree of 16S rRNA gene. Other pathogens in this column also represented the corresponding gene sequences in the phylogenetic tree of 16S rRNA gene.

^#^ *Ehrlichia ruminantium* 16S rRNA gene sequence (NR 074513).

Table S6 Estimates of evolutionary divergence over sequence pairs between groups of *gltA* gene

| Group | P distance |  |  |  |  |  |  |  |
| --- | --- | --- | --- | --- | --- | --- | --- | --- |
| Group Ⅱ |  |  |  |  |  |  |  |  |
| Group Ⅰ | 0.2459958932 |  |  |  |  |  |  |  |
| *A. ovis^@^* | 0.0071868583 | 0.2521560575 |  |  |  |  |  |  |
| *A. capra* | 0.2464065708 | 0.0012320329 | 0.2525667351 |  |  |  |  |  |
| *Anaplasma* sp. | 0.2464065708 | 0.0012320329 | 0.2525667351 | 0.0000000000 |  |  |  |  |
| *A. phagocytophilum* | 0.3798767967 | 0.3568788501 | 0.3757700205 | 0.3572895277 | 0.3572895277 |  |  |  |
| Outgroup ^$^ | 0.6201232033 | 0.6151950719 | 0.6160164271 | 0.6160164271 | 0.6160164271 | 0.6036960986 |  |  |
| *A. platys* | 0.3997262149 | 0.3930184805 | 0.4038329911 | 0.3942505133 | 0.3942505133 | 0.4045174538 | 0.6173853525 |  |
| *A. marginale* | 0.1221765914 | 0.2492813142 | 0.1232032854 | 0.2505133470 | 0.2505133470 | 0.3572895277 | 0.6290212183 | 0.3764544832 |

*^@^* All *A. ovis* gene sequences in the phylogenetic tree of *gltA* gene. Other pathogens in this column also represented the corresponding gene sequences in the phylogenetic tree of *gltA* gene.

^$^ Ehrlichia sp. *gltA* gene sequence (MK294041).

Table S7 Estimates of evolutionary divergence over sequence pairs between groups of *groEL* gene

| Group | P distance | |  |  |  |  |  |  |
| --- | --- | --- | --- | --- | --- | --- | --- | --- |
| This study^a^ |  |  | |  |  |  |  |  |
| *A. phagocytophilum*^b^ | 0.1616 |  | |  |  |  |  |  |
| *Anaplasma* sp. | 0.0911 | 0.1784 | |  |  |  |  |  |
| *A. ovis* | 0.2016 | 0.2165 | | 0.1997 |  |  |  |  |
| *A. marginale* | 0.1863 | 0.1799 | | 0.1966 | 0.0915 |  |  |  |
| *A. centrale* | 0.2022 | 0.1951 | | 0.2180 | 0.0915 | 0.0457 |  |  |
| *A. capra* | 0.2010 | 0.2083 | | 0.2048 | 0.1667 | 0.1636 | 0.1616 |  |
| Outgroup^c^ | 0.2493 | 0.2287 | | 0.2668 | 0.2713 | 0.2591 | 0.2591 | 0.2683 |

^a^ All the *groEL* gene sequences obtained in this study.

^b^ All the *A. phagocytophilum* gene sequences in the phylogenetic tree of *groEL* gene. Other pathogens in this column also represented the corresponding gene sequences in the phylogenetic tree of *groEL* gene.

^c^ *Ehrlichia ruminantium* gene sequence (GQ457107).

Table S8 Estimates of evolutionary divergence over sequence pairs between groups of *msp4* gene

| Group | P disatance | |  |  |  |  |  |  |  |
| --- | --- | --- | --- | --- | --- | --- | --- | --- | --- |
| *Anaplasma* sp.^d^ |  |  | |  |  |  |  |  |  |
| *A. centrale* | 0.209 |  | |  |  |  |  |  |  |
| *A. phagocytophilum* | 0.372 | 0.369 | |  |  |  |  |  |  |
| *A. ovis* | 0.154 | 0.178 | | 0.362 |  |  |  |  |  |
| *A. marginale* | 0.171 | 0.175 | | 0.367 | 0.104 |  |  |  |  |
| Group ⅰ | 0.351 | 0.337 | | 0.248 | 0.336 | 0.350 |  |  |  |
| Group ⅱ | 0.317 | 0.318 | | 0.262 | 0.328 | 0.324 | 0.256 |  |  |
| *A. capra* | 0.117 | 0.238 | | 0.372 | 0.238 | 0.249 | 0.361 | 0.314 |  |
| Outgroup^e^ | 0.637 | 0.661 | | 0.615 | 0.609 | 0.628 | 0.637 | 0.606 | 0.639 |

^d^ All the *Anaplasma* sp. gene sequences in the phylogenetic tree of *msp4* gene. Other pathogens in this column also represented the corresponding gene sequences in the phylogenetic tree of *msp4* gene.

^e^ *Ehrlichia ruminantium* gene sequence (BDDN01000174).

**Reference**

Bown, K.J., Lambin, X., Ogden, N.H., Petrovec, M., Shaw, S.E., Woldehiwet, Z., et al. (2007). High-resolution genetic fingerprinting of European strains of *Anaplasma phagocytophilum* by use of multilocus variable-number tandem-repeat analysis. *J Clin Microbiol* 45(6)**,** 1771-1776. doi: 10.1128/JCM.00365-07.

de la Fuente, J., Massung, R.F., Wong, S.J., Chu, F.K., Lutz, H., Meli, M., et al. (2005). Sequence analysis of the msp4 gene of *Anaplasma phagocytophilum* strains. *J Clin Microbiol* 43(3)**,** 1309-1317. doi: 10.1128/JCM.43.3.1309-1317.2005.

Li, H., Jiang, J.F., Liu, W., Zheng, Y.C., Huo, Q.B., Tang, K., et al. (2012). Human infection with Candidatus *Neoehrlichia mikurensis*, China. *Emerg Infect Dis* 18(10)**,** 1636-1639. doi: 10.3201/eid1810.120594.

Li, H., Zheng, Y.C., Ma, L., Jia, N., Jiang, B.G., Jiang, R.R., et al. (2015). Human infection with a novel tick-borne *Anaplasma* species in China: a surveillance study. *Lancet Infect Dis* 15(6)**,** 663-670. doi: 10.1016/S1473-3099(15)70051-4.

Wen, B., Jian, R., Zhang, Y., and Chen, R. (2002). Simultaneous detection of *Anaplasma marginale* and a new *Ehrlichia* species closely related to *Ehrlichia chaffeensis* by sequence analyses of 16S ribosomal DNA in *Boophilus microplus* ticks from Tibet. *J Clin Microbiol* 40(9)**,** 3286-3290. doi: 10.1128/jcm.40.9.3286-3290.2002.

Zhang, W.J. (2015). The genetic characteristics of *Anaplasma phagocytophilum* from sheep and goats. Master degree, Henan Agricultural University.
